# Insights into Tissue-Specific Specialized Metabolism in Wampee (*Clausena lansium* (Lour.) Skeels) Varieties

**DOI:** 10.3390/foods13193092

**Published:** 2024-09-27

**Authors:** Ran Zhang, Junjie Zhou, Xiaoxuan Zhang, Huanteng Hou, Xianqing Liu, Chenkun Yang, Shuangqian Shen, Jie Luo

**Affiliations:** 1 School of Breeding and Multiplication (Sanya Institute of Breeding and Multiplication), Hainan University, Sanya 572025, China; zhangran@hainanu.edu.cn (R.Z.); sand_zhou@hainanu.edu.cn (J.Z.); 17868912065@139.com (X.Z.); naturehht@foxmail.com (H.H.); liuxq@hainanu.edu.cn (X.L.); yangchenkun@yzwlab.cn (C.Y.); shenshuangqian@yzwlab.cn (S.S.); 2 Yazhouwan National Laboratory, Sanya 572025, China

**Keywords:** *Clausena lansium* (Lour.) Skeels, metabolic profiling, biomarker, spatial distribution, MALDI-MSI, nutritional breeding

## Abstract

Wampee (*Clausena lansium* (Lour.) Skeels) has natural bioactive components with diverse health benefits, but its detailed metabolism and tissue distribution are not fully understood. Here, widely targeted metabolomics analysis methods were employed to analyze the wampee fruit (peel, pulp, and seed) of 17 different varieties. A total of 1286 metabolites were annotated, including lipids, flavonoids, polyphenols, carbazole alkaloids, coumarins, and organic acids, among others. The quantitative analysis and matrix-assisted laser desorption/ionization–mass spectrometry imaging (MALDI-MSI) analysis indicated remarkable variations in metabolite categories and content in the peel, pulp, and seed of wampee fruit. Additionally, the difference analysis found that the metabolic components of peel contributed dominantly to the differences among varieties, and 7 potential biomarkers were identified. In this study, a comprehensive metabolome landscape of wampee fruit was established, which provided important information for the isolation and identification of functional components, food industry application, and nutritional improvement breeding.

## 1. Introduction

Wampee (*Clausena lansium* (Lour.) Skeels) is a plant native to southern China and belongs to the Rutaceae family. It has been documented as a medicinal and edible fruit in the classics of traditional Chinese medicine in ancient China. In addition to its edible pulp, its leaves, fruit stones, and peels can also serve as potential medicinal materials with effects such as diuresis and detumescence, promoting fluid production and quenching thirst, clearing heat, and relieving cough. Therefore, it is known as the “treasure of fruits” [1]. The fruit of wampee is sweet and sour with a distinctive aroma, rich in active substances that have been widely used in the food processing industry. Apart from being consumed fresh or for medicinal purposes, it is also transformed into preserved fruits, fruit juice, pastries, etc. Currently, wampee is widely cultivated in tropical regions such as southern China, India, Australia, Thailand, and Vietnam.

For plants with medicinal and nutritional value, tissue differential analysis is indispensable, which not only helps to reveal the nutritional and medicinal value of different tissues but also helps to distinguish the precise distribution of important categories of bioactive compounds in different tissues. Researchers have examined the metabolites in different tissues of medicinal plants such as Panax notoginseng and Ginseng [2,3] and found that different types of ginsenosides accumulate in various tissues, most of which play roles in anti-inflammatory, anti-cancer, antioxidant, and immune regulation. In addition to these, metabolites in different tissues of olive (*Olea europaea* L.), *Eucommia ulmoides* Oliver, and blackberry have also been characterized for their differential profiles [4,5,6], and these studies are of significant importance for guiding the selection of edible parts and precision breeding. Metabolites in different tissues of wampee possess a variety of nutritional and medicinal values, such as antioxidant, antibacterial, anti-tumor, liver-protecting with lipid-lowering effects, and blood sugar-lowering activities [7,8,9,10,11,12]. So far, natural products such as carbazole alkaloids, coumarins, amide alkaloids, terpenes, and flavonoids have been successfully isolated and identified from different tissues of wampee [11,13]. However, the above studies mostly focused on the detection and analysis of various tissues and organs, such as leaves, stems, and fruits, and focused on the detection, separation, and purification analysis of extracts of a single component [7,9,14]. Due to the small number of metabolites detected and the lack of precise quantification, it is not possible to fully characterize the wampee fruit and effectively identify the differences between its fine tissues. By constructing the metabolome panorama of multiple tissues of various varieties of wampee fruit and analyzing the key metabolite biomarkers, it will greatly enrich the cognition and evaluation of the nutritional and health value of wampee fruit.

With the rapid development of metabolomics [15,16,17,18], detection instruments have also become increasingly diverse and sophisticated, such as liquid chromatography–mass spectrometry (LC–MS), gas chromatography-mass spectrometry (GC-MS), and matrix-assisted laser desorption/ionization mass spectrometry imaging (MALDI-MSI), providing opportunities for comprehensive detection of wampee metabolites. Due to the low sensitivity of non-targeted detection and the low throughput of targeted detection, a widely targeted metabolomics analysis method that combines the high throughput of non-targeted analysis with the high sensitivity of targeted analysis has become a powerful tool in metabolomics [19]. Due to the complexity of metabolites and the diversity of their chemical structures, it is not possible to analyze all metabolites simultaneously using a single platform. Currently, the metabolites detected in plants may account for less than 10% of their total quantity [20], but widely targeted metabolites aim to accurately and effectively detect as many metabolites as possible [19]. At present, a variety of plants, such as rice [17], Qingke [21], maize [22], tomato [23], pummelo [24], and citrus [25], among others, have been comprehensively characterized by widely targeted metabolites, and further studies have been conducted based on this combination with genome and transcriptome. Metabolomics plays an indispensable role in elucidating the dynamic changes of a series of biological activities as well as the comprehensive characterization of species [16]. In the field of multi-tissue metabolomics research in plants, this analytical strategy has also been used to establish a metabolite profiling database covering the entire growth period of rice, including eight main tissue organs (coleoptile, radicle, leaf, leaf sheath, stem, root, panicle, and seed). By analyzing the differences in metabolic signals among various tissues, it has been found that there are multiple metabolic signals that are specifically present in each tissue [17].

Yongxing Town of Haikou City, Hainan Province, is one of the world’s original birthplaces and a gene bank for the germplasm resources of wampee. The wild wampee forest in Yongxing has a long history of growth. The germplasm resources of Yongxing wampee are abundant and unique, and they have cultivated some of the world’s unique local varieties of wampee, such as Yongxing seedless wampee, Yongxing big chicken heart wampee, Yongxing small chicken heart wampee, and so on. In order to systematically clarify the differences between the three tissues (peel, pulp, and seed) of the wampee fruit, this study adopted targeted and non-targeted metabolomics methods. to detect and comprehensively analyze various categories of metabolites from 17 varieties collected and established a metabonomics database. Combined with quantitative data and spatial metabolomics detection, the differences of metabolites and metabolic networks specifically accumulated in three tissues were analyzed. In addition, key biomarkers were screened for variety differentiation. This study provided a theoretical basis for fine processing and metabolome-assisted breeding of wampee.

## 2. Materials and Methods

### 2.1. Plant Materials and Sample Preparation

Samples were collected from Yongxing Town in Haikou, one of the original habitats of the wampee fruit in the world. During the ripening period of the wampee fruit, plants with consistent phenotypes were selected for sampling. Each sample was a mixture of fruits from three different plants, which was then combined to serve as a sample for one biological replicate. Each material was set with three independent biological replicates. Detailed information on sample collection and photos of the fruits are displayed in Supplemental Table S1 and Figure S1. The ripe fruits were washed with sterile deionized water, and the peel was cut open with a sterilized scalpel. The peel and pulp were quickly separated and immediately placed in liquid nitrogen, followed by storage at −80 °C, and then subjected to vacuum freeze-drying. After freeze-drying, the pulp and seed were further separated.

The samples were ground using a grinding apparatus (MM 400; Retsch, Haan, Germany) with zirconia beads at 30 Hz for 1 min. One hundred milligrams of the powder was weighed and extracted with 0.8 mL of 70% methanol aqueous solution (methanol: H_2_O, 7:3, *v*/*v*) containing 10 ppb lidocaine, and was incubated overnight at 4 °C. After centrifugation at 12,000× *g* for 10 min, the supernatant was filtered (SCAA-104, pore size 0.22 µm; ANPEL, Shanghai, China, (http://www.anpel.com.cn/ (accessed on 25 June 2023)) and then analyzed by LC–MS [19]. To investigate the inter-tissue differences in metabolites, samples from the same tissue of different varieties were mixed for untargeted metabolomics analysis. The samples were equally mixed into multiple quality control samples to check the stability of the instrument.

### 2.2. Reagents and Standards

HPLC-grade acetonitrile, acetic acid, and methanol were purchased from Unity™ Lab Services (Thermo Fisher Scientific, Waltham, MA, USA); water was purified using a Thermo Scientific LabTower EDI 15 purification system (Thermo Fisher Scientific, Waltham, MA, USA). Lidocaine, used as an internal standard in this study, was obtained from Tokyo Chemical Industry Co., Ltd., Tokyo, Japan (https://www.tokyochemical.lookchem.com/ (accessed on 15 October 2022)). Carboxymethyl cellulose (CMC) and N-(1-naphthyl) ethylenediamine dihydrochloride (NEDC) were purchased from Shanghai Macklin Biochemical Technology Co., Ltd., Shanghai, China (https://www.macklin.cn/ (accessed on 7 April 2024)), and 2,5-Dihydroxybenzoic acid (DHB) was purchased from Shanghai Yuanye Bio-Technology Co., Ltd., Shanghai, China (https://www.shyuanye.com/ (accessed on 7 April 2024)).

The imperatorin, myricitrin, and scopoletin used in this study were provided by ChemFaces, Wuhan, China (http://www.chemfaces.com/ (accessed on 18 September 2023)). Catechin, Gallocatechin, and LysoPC (18:1) were supplied by Shanghai Yuanye Bio-Technology Co., Ltd., Shanghai, China (https://www.shyuanye.com/ (accessed on 18 September 2023)). All standards were prepared by dissolving in methanol to make the stock solutions, which were then diluted to the appropriate concentration using a 70% methanol aqueous solution (methanol: H_2_O, 7:3, *v*/*v*) for detection on the LC–MS. All standard stock solutions were stored at −80 °C in the dark.

### 2.3. LC–MS/MS Analysis of Metabolites

Samples were analyzed using a targeted method combined with a non-targeted metabolomics analysis method based on ultra-performance liquid chromatography-tandem mass spectrometry (UPLC–MS/MS) [17,19]. Non-targeted analysis was performed using an HPLC-ESI-Q-Exactive-MS/MS (Thermo Scientific Q-Exactive, Thermo Fisher Scientific, Waltham, MA, USA). Mass spectrometric detection used a HESI ion source for non-targeted metabolites profiling in full MS and ddMS2 mode to obtain the data, including the accurate masses, MS/MS fragments, and retention times. The recording conditions were as follows: source capillary, 3.2 kV; sheath gas flow rate, 7 psi; source temperature, 400 °C; scan range, *m*/*z* 100–1000. Targeted analysis of metabolites was conducted in multiple reaction monitoring (MRM) mode using an LC-ESI-Q TRAP-MS/MS (AB Sciex 6500, Applied Biosystems, Woburn, MA, USA). The ESI source operation parameters were as follows: temperature of 500; GSI, GSII, and CUR of 50, 60, and 35 psi, respectively; and IS of 5500 V in positive mode or −4500 V in negative mode; the collision gas was high. The total sMRM cycle time was set to 1.0 s, whereas the dwell time for each MRM transition was automatically adjusted according to the total cycle time, ensuring that at least 10 points were obtained for each peak. Data acquisition and analysis were carried out using SCIEX OS 3.1.5.3945 software (AB Sciex, Framingham, MA, USA). The quantification of metabolites was performed by calculating the peak area and comparing it to the standard curve drawn by the lidocaine standard.

### 2.4. MALDI-MSI Analysis of Metabolites

Wampee fruit was embedded in 2% carboxymethyl cellulose (CMC) and rapidly frozen. Subsequently, the frozen tissue was sectioned into 25-micrometer-thick slices at −20 °C using a Leica CM1950 cryostat (Leica Microsystems GmbH, Wetzlar, Germany). The sections were mounted onto indium tin oxide (ITO)-coated conductive glass slides (Bruker Daltonics, Bremen, Germany). Matrix solutions of 15 mg/mL DHB (2,5-dihydroxybenzoic acid, positive model) and 7 mg/mL NEDC (N-(1-naphthyl)ethylenediamine dihydrochloride, negative model) were applied using the HTX TM-Sprayer (HTX Technologie, CA, USA), following the standard protocol provided by the manufacturer for matrix solution preparation and application. MSI experiments were conducted using a timsTOF fleX MALDI-TOF/TOF (Bruker Daltonics, Bremen, Germany). The spatial resolution was set to 50 μm, with a mass range of *m*/*z* 20–1300. Data acquisition was performed using timsControl 3.1 and flexImaging 7.0 (Bruker Daltonics, Bremen, Germany), and data visualization was carried out with the SCiLS Lab 2023b 11.01.14623 software.

### 2.5. Classification and Identification of Metabolites

For non-targeted data, the Thermo Compound Discoverer 3.3 software was used for data extraction. By extracting molecular ions and fragment ions, an MS2 spectral tag (MS2T) library was created, which was then annotated based on the accurate *m/z* value, retention time (RT), and fragmentation pattern. Following the approach pioneered in plants by Morreel et al. [26], these features were used to screen the data in the literature and database. The annotated metabolites were further identified with the help of available standards. For targeted data, SCIEX OS 3.1.5.3945 software (AB Sciex, Framingham, MA, USA) was used to align peaks by retention time (RT), and the quantification of metabolites was completed by calculating the peak area.

### 2.6. Statistical Data Analysis

For metabolomics data analysis, principal component analysis (PCA) was conducted using the OmicStudio tools (www.omicstudio.cn/tool (accessed on 27 June 2024)). Hierarchical cluster analysis and sample repeatability assessment were performed using the R software (http://www.r-project.org/ (accessed on 13 July 2024)). Data were log2-transformed to improve normalization, followed by sample repeatability assessment. *Z* score normalization was applied for data normalized, which was then followed by hierarchical cluster analysis and PCA. For the comparison of absolute metabolite content across the three tissues, two-tailed Student’s *t*-tests were used, and *p* ≤ 0.05 was considered significant. Orthogonal Projection to Latent Structures Discriminant Analysis (OPLS-DA) was conducted using the Metware Cloud, a free online platform for data analysis (https://cloud.metware.cn/#/home (accessed on 27 June 2024)). The data used for computation and analysis in this study were the average values of three biological replicates.

## 3. Results

### 3.1. Non-Targeted Metabolomics Analysis in Wampee Fruit

In order to comprehensively evaluate and characterize the differences in metabolic profiles of wampee fruit, we collected 17 wampee fruit materials from different natural villages in Yongxing Town, Haikou, and performed metabolomic analysis based on non-targeted HPLC-TOF-MS.

**Figure 1:**
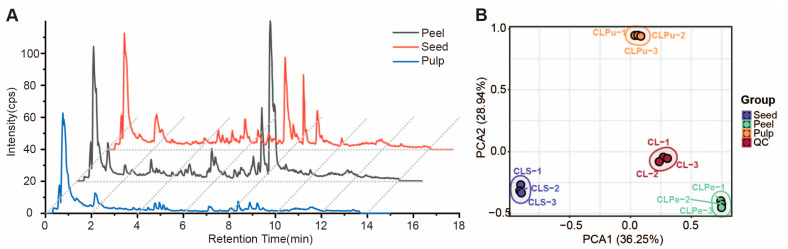
Analysis of metabolic variation in three tissues using ESI − Q − Exactive LC − MS/MS. (**A**) Total ion chromatography of metabolites in three tissues of the wampee fruit. (**B**) Principal component analysis (PCA) of total ion chromatography results for three tissues of the wampee fruit.

To gain a comprehensive understanding of the changes in metabolites between different tissues and varieties of wampee fruit, we tested and analyzed tissue mixtures of different varieties, detecting nearly 30,000 mass spectrometry signals in three mixed tissues. The metabolic profile signals in the total ion current chart showed significant differences among the three tissues: peel, pulp, and seed (Figure 1A), especially between 7 and 10 min, which may be due to some lipophilic metabolites. Additionally, principal component analysis (PCA) was conducted on the three tissues based on the metabolic signals obtained from the non-targeted analysis. The PCA showed that component 1 and component 2 accounted for 36.25% and 28.94% of the variation, respectively (Figure 1B). Components 1 and 2 successfully separated the three tissues, indicating significant differences in metabolite levels among them. Furthermore, the non-targeted metabolomics analysis of different tissues helps to discover new metabolites that may not exist in traditional single tissues, suggesting that this strategy can greatly facilitate the establishment of a comprehensive metabolite database.

### 3.2. Metabolite Identification/Annotation Based on Non-Targeted Metabolic Analysis

In this study, to gain a deeper understanding of metabolites in different tissues, we used Thermo Compound Discoverer 3.3 software for data extraction from non-targeted metabolomic profiling data. After filtering for signal-to-noise ratio and deduplication, there were 12,000 metabolic signals. An MS2 spectral tag (MS2T) library was established using the obtained metabolic signals, and the MS2T spectral tags were annotated based on accurate *m*/*z* values, retention time (RT), and fragmentation patterns. These characteristics were used to screen in literature and databases and further confirmed using commercially available standards. Additionally, by applying the rules of compound fragmentation, similar compounds were inferred and identified.

**Figure 2:**
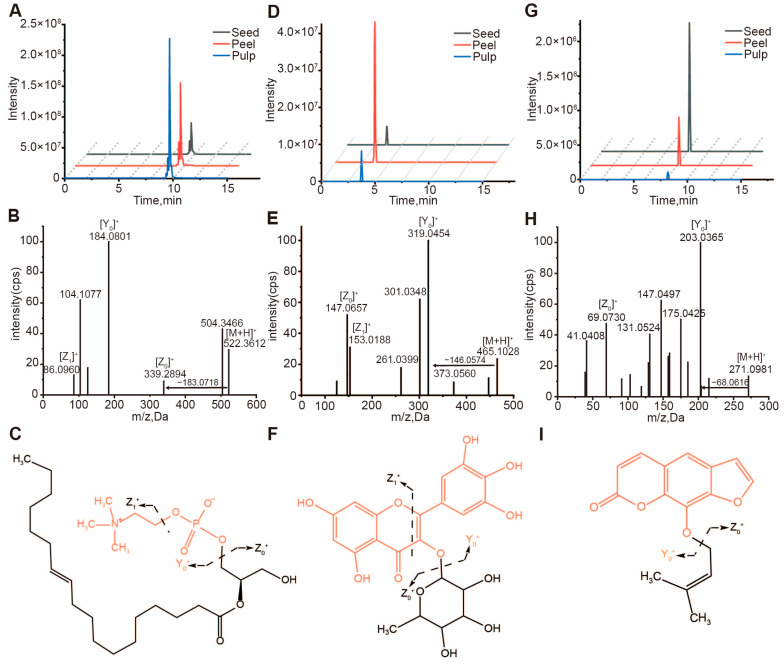
Detection and identification of specific LysoPC (18:1), myricitrin, and imperatorin metabolite signals by Q − Exactive LC − MS/MS. (**A**) EIC (extracted ion chromatogram) of CLP0940 at 9.86 min. (**B**) MS/MS spectra of CLP0940 at *m/z* 522.3612, and the metabolite was identified as LysoPC (18:1). (**C**) The molecular structure of the LysoPC (18:1) and its general fragmentation rules. (**D**) EIC (extracted ion chromatogram) of CLP0905 at 3.66 min. (**E**) MS/MS spectra of CLP0905 at *m/z* 465.1028, and the metabolite was identified as myricitrin. (**F**) The molecular structure of the myricitrin and its general fragmentation rules. (**G**) EIC (extracted ion chromatogram) of CLP0658 at 8.21 min. (**H**) MS/MS spectra of CLP0658 at *m/z* 271.0981, and the metabolite was identified as imperatorin. (**I**) The molecular structure of the imperatorin and its general fragmentation rules.

Lysophosphatidylcholine (LPC) and phosphatidylcholine (PC) are important subclasses of glycerophospholipids. LPC and PC produce characteristic phosphate choline product ions at *m/z* 184.1 and then lose N(CH_3_)_3_^+^ and H_2_O to form fragments at *m/z* 125.0 and 86.1, respectively. For instance, the compound CLP0940 was detected at a retention time (RT) of 9.86 min (Figure 2A). CLP0940 generated a precursor ion [M+H]^+^ at *m/z* 522.3612, and the mass spectrum showed a characteristic fragment Y_0_^+^ ion at *m/z* 184.0801 for CLP0940, along with fragments of *m/z* 124.9998 and 86.0960, which indicated the presence of a phosphate choline skeleton. The Z_0_^+^ ion at *m/z* 339.2894 [M+H-183]^+^ corresponded to the losses of a phosphate choline moiety. These observations allowed us to characterize CLP0940 as LysoPC (18:1) (Figure 2B). By comparing the secondary mass spectrum of CLP0940 with that of the LysoPC (18:1) standard, our annotation was ultimately confirmed to be correct, with the structure and main fragmentation pathways of LysoPC (18:1) shown in Figure 2C. Based on similar fragmentation patterns, we annotated 10 LPCs and found that LPCs are more abundantly accumulated in the pulp.

Flavonoid compounds are a class of secondary metabolites widely distributed in plants, including several subclasses such as anthocyanins, flavonols, flavones, flavanols, flavanones, catechins, etc. Glycosylation is a common modification of flavonoids that produces characteristic fragments of flavonoid aglycone ions, characteristic product ions of flavonoid aglycones, and modified ion fragments. For example, a compound CLP0905 (Figure 2D) was detected at a retention time (RT) of 3.66 min, where CLP0905 generated a precursor ion [M+H]^+^ at *m/z* 465.1028. The mass spectrum showed that CLP0905 has a characteristic fragment Y_0_^+^ ion at *m/z* 319.0454 and characteristic fragments at *m/z* 153.0188 and 261.0399, indicating that its flavonoid aglycone is myricetin. The Z_0_^+^ ion at *m/z* 147.0657 [M+H-318]^+^ corresponds to the loss of the rhamnoside modification. These observations allowed us to characterize CLP0905 as myricetin 3-O-rhamnoside, which is myricitrin (Figure 2E). By comparing the MS/MS spectrum of CLP0905 with that of the myricitrin standard, our annotation was ultimately confirmed to be correct, and the structure and main fragmentation pathways of myricitrin are shown in Figure 2F. Based on similar fragmentation patterns, we annotated 14 flavonoid glycosides and found that they accumulate more in the fruit peel. Catechins are an important subclass of flavonoids. Catechin subclasses produce a fragment Y_0_^+^ ion at *m/z* 139.04. Based on this, by comparing with the standard, we identified catechin and (+)-gallocatechin (GC), and their extracted ion current maps, MS/MS spectra, and fragmentation patterns are shown in Supplemental Figure S2A–F, and it was found that they also accumulate more in the fruit peel.

Coumarin compounds are a class of metabolites with broad pharmacological activities in the Clausena genus, which are categorized into different types based on their structure, such as simple coumarins, furanocoumarins, and pyranocoumarins. Furanocoumarins produce a characteristic product ion at *m/z* 203.04. For instance, the compound CLP0658 was detected at a retention time (RT) of 8.21 min (Figure 2G). CLP0658 generated a precursor ion [M+H]^+^ at *m/z* 271.0981, and the mass spectrum showed a characteristic fragment Y_0_^+^ ion for CLP0658 at *m/z* 203.0365, along with a Z_0_^+^ ion at *m/z* 69.0730 [M+H-203]^+^ corresponding to the loss of the furanocoumarin substituent. These observations allowed us to characterize CLP0658 as imperatorin (Figure 2H). By comparing the secondary mass spectrum of CLP0658 with that of the imperatorin standard, the annotation was ultimately confirmed to be correct, with the structure and main fragmentation pathways of Imperatorin shown in Figure 2I. Based on similar fragmentation patterns, we annotated six furanocoumarins and found that they are more abundantly accumulated in the seeds and peels of some varieties. Simple coumarins produce a characteristic product ion at *m/z* 163.04. For example, the compound CLP0720 was detected at an RT of 3.81 min (Supplemental Figure S2G). CLP0720 generated a precursor ion [M+H]^+^ at *m/z* 193.0528, and the mass spectrum showed a characteristic fragment Y_0_^+^ ion for CLP0720 at *m/z* 178.0266, along with the characteristic coumarin product ion Z_0_^+^ at m/z 163.04. These observations led us to infer that it might be a coumarin metabolite but not a typical monoterpene substitution or isoprenyl substitution; instead, it is a simpler form of substitution. By comparing with the standard, we confirmed this metabolite as scopoletin (Supplemental Figure S2H), with its structure and main fragmentation pathways shown in Supplemental Figure S2I.

**Figure 3:**
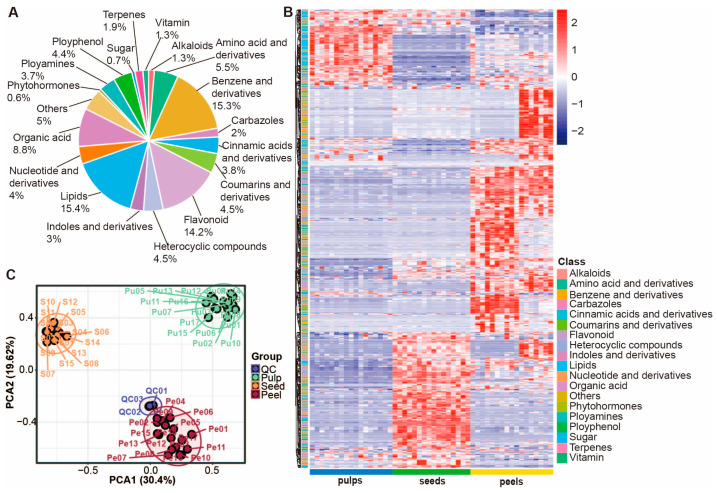
Summary of metabolic profiling and tissue variability analysis. (**A**) Categorical pie chart of the 1286 annotated metabolites. (**B**) Hierarchically clustered heatmap of the 1286 annotated metabolites from 50 wampee samples. (**C**) PCA results for the metabolome data from 50 wampee samples.

By summarizing and annotating other types of metabolites in this manner, we have identified a total of 1286 specific metabolites, mainly consisting of flavonoids, lipids, benzene and its derivatives, and organic acids, with their classification and proportion as shown in Figure 3A, that is, 14.2% flavonoids, 15.4% lipids, 15.3% benzene and its derivatives, 8.8% organic acids, etc. This study is the first to quantify a large number of lipids, phenolic amides, coumarins, and terpenoids in multiple tissues of wampee fruit, such as some glycerophospholipids, dCMP, myricitrin, sudachiin C, lansamide 4, and other compounds that have been identified for the first time in wampee fruit.

### 3.3. Targeted Metabolomics Analysis in Wampee Fruit

To analyze the accumulation patterns of metabolites in different varieties and tissues of wampee fruit, we conducted targeted quantitative detection of 1286 metabolites in three tissues of 17 varieties of wampee fruits using LC–MS. First, a correlation analysis was performed on the results, which revealed good repeatability among the three replicates, a high correlation within the tissue, and reliable metabolomics detection results, indicating that the data are suitable for further analysis (Supplemental Figure S3A). Subsequently, we performed PCA and found that samples from the same tissue clustered together, indicating that tissue specificity is greater than variety specificity (Figure 3C). Next, we conducted hierarchical cluster analysis on the content of 1286 metabolites in 50 different samples. Hierarchical cluster analysis groups data by constructing a multi-level nested classification system, which allows different compounds to be clustered together due to their similar accumulation patterns in different varieties and tissues. A heatmap can more clearly and intuitively show the differential accumulation of substances. The results showed that there is a significant tissue difference in metabolite accumulation, with higher lipid accumulation in the pulp, higher accumulation of flavonoids in the peel, and a rich accumulation of various metabolites in the seed, such as flavonoids, coumarins, and carbazole alkaloids. In addition, there are considerable differences in metabolites between different varieties in the peel, such as lipids, coumarins, and terpenes (Figure 3B).

To compare the accumulation patterns of metabolites in each tissue, we generated a Venn diagram by screening differential metabolites, which shows the intersection and differential accumulation of metabolites between different tissues (Supplemental Figure S3B). Consistent with the hierarchical clustering results, the number of metabolites in the peel is the highest. Furthermore, compared to the pulp, the peel is richer in flavonoids, carbazole alkaloids, quinic acid and its derivatives, and indole compounds (Supplemental Figure S4A). Compared to the seed, the peel is richer in glycerophospholipids, quinic acid and its derivatives, and indole compounds (Supplemental Figure S4B). When comparing the pulp to the seed, the pulp is richer in glycerophospholipids, while the seed is richer in anthocyanins, flavonoids, and terpenes (Supplemental Figure S4C). Some information on differential metabolites can be found in Supplemental Table S2.

### 3.4. Tissue and Variety Analysis of Wampee Fruit Metabolites Based on LC–MS

#### 3.4.1. Accumulation Patterns of Metabolites in the Pulps of Wampee

**Figure 4:**
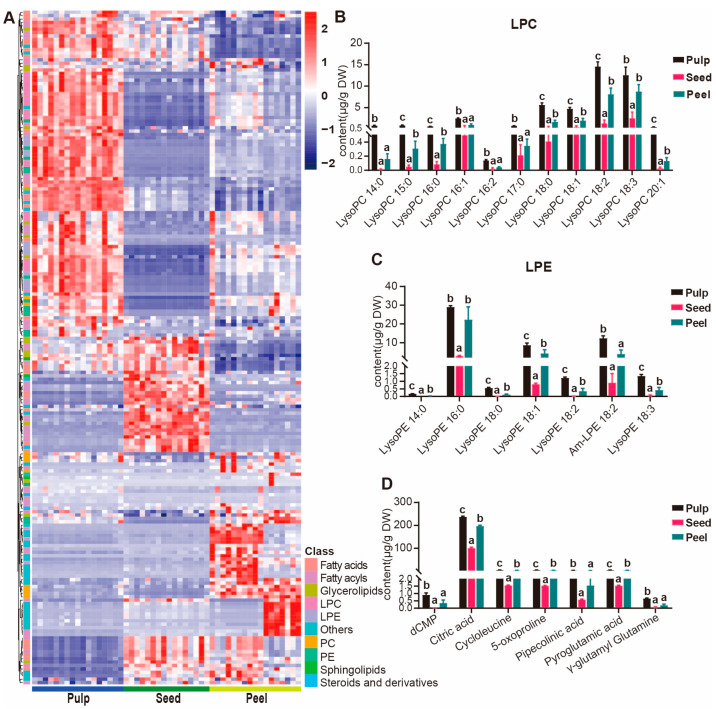
Distribution of lipids in different wampee fruit tissues. (**A**) Hierarchically clustered heatmap of the 198 lipid metabolites from 50 wampee samples. (**B**,**C**) Bar plots showing the contents of LPC and LPE content of various wampee fruit tissues, respectively. (**D**) Bar plots showing the content of substances that accumulate more in the pulps of wampee compared to other tissues, such as organic acids and amino acids. When using the abc notation to indicate significant differences, the same letter indicates no significant difference between groups (*p* > 0.05), and different letters indicate significant differences (*p* < 0.05).

Based on the hierarchical clustering analysis results of the 1286 metabolites, we found that the pulp has the richest accumulation of lipids. In this study, lipids were categorized into subclasses such as fatty acids, fatty acyls, glycerolipids, lysophosphatidylcholine (LPC), lysophosphatidylethanolamine (LPE), phosphatidylcholine (PC), phosphatidylethanolamine (PE), sphingolipids, and steroids. Therefore, we conducted hierarchical cluster analysis on the 198 detected lipid subclass metabolites and found that the accumulation of glycerophospholipids in the pulp is relatively abundant (Figure 4A). At the same time, we found that some lipids have differential accumulation in the peel among different varieties, such as penidienone, 2,4,6-octatriyn-1-ol, pyreudione B, and diatretyne 3. We further conducted hierarchical cluster analysis on the glycerophospholipids and found that LPC, LPE, and PE mainly accumulate in the pulp, while PC mainly accumulates in the peel (Supplemental Figure S5A). Further research on the absolute content of the four subclasses of glycerophospholipids showed that LPC, LPE, and PE have significantly higher accumulation levels in the pulp, followed by the peel (Figure 4B,C and Figure S5C). In contrast, the accumulation of PC in the pulp is lower than in the peel (Supplemental Figure S5B). LPC and LPE have emulsifying effects, which can promote the digestion and absorption of lipids in the human body and improve the efficiency of nutritional utilization [27], which is consistent with the rich nutritional value of the pulp. In addition to this, the pulp is also rich in organic acids, such as citric acid, and amino acids, such as 5-oxoproline and gamma-glutamyl glutamine (Figure 4D).

#### 3.4.2. Accumulation Patterns of Metabolites in the Peels of Wampee

**Figure 5:**
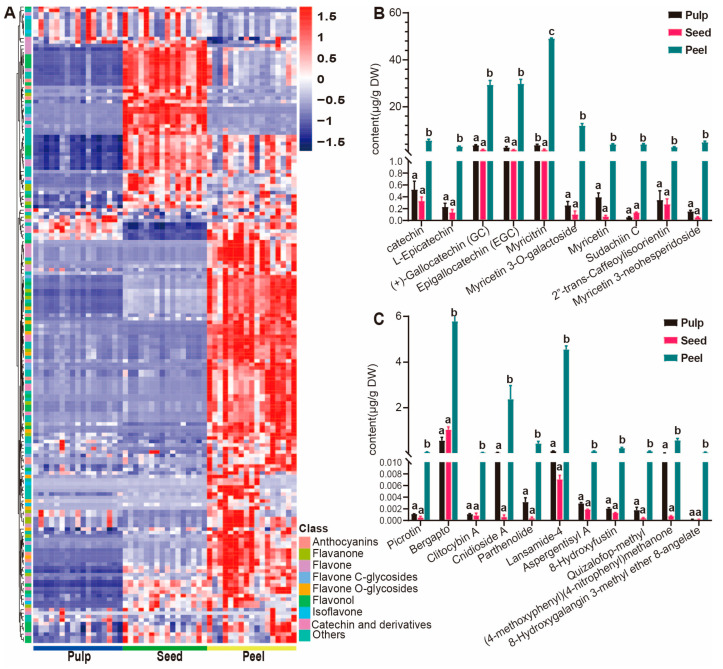
Distribution of flavonoid in different wampee fruit tissues. (**A**) Hierarchically clustered heatmap of the 182 flavonoid metabolites from 50 wampee samples. (**B**) Bar plots showing the flavonoids that are more abundant in the peel of wampee. (**C**) Bar plots showing the content of substances that accumulate more in the peels of wampee compared to other tissues, such as terpenes and polyphenol. When using the abc notation to indicate significant differences, the same letter indicates no significant difference between groups (*p* > 0.05), and different letters indicate significant differences (*p* < 0.05).

Based on the hierarchical clustering analysis results of the 1286 metabolites, we found that the accumulation of flavonoids in the peel is the most significant. Therefore, we conducted hierarchical cluster analysis on the 182 detected flavonoid metabolites and found that flavonoids are most abundantly accumulated in the peel, followed by the seed (Figure 5A). To explore the specific accumulation of each subclass within flavonoids, we performed hierarchical cluster analysis on the main subclasses of flavonoids and found that the number of metabolites such as anthocyanins, flavones, and flavonols that are specifically accumulated in the peel and seed is considerable, but the accumulation patterns differ (Supplemental Figure S6A–C). At the same time, most of the catechin, flavonoid glycosides, and flavanone metabolites accumulate in the peel, with a smaller portion accumulating in the seed (Supplemental Figure S6D–F). Additionally, based on the absolute content, we screened metabolites that are specifically accumulated in the peel with a content greater than 3 micrograms per milliliter and have a difference of more than 10 times compared to the seed (Figure 5B), such as catechin, L-epicatechin, (+)-gallocatechin (GC), epigallocatechin (EGC), and other catechin metabolites, as well as myricetin, sudachiin C, myricetin 3-neohesperidoside, and other flavonoid glycosides, which play an important role in health maintenance and promotion [28], including antioxidant [29], regulation of blood lipid metabolism, anti-inflammatory [30], and prevention of cardiovascular and cerebrovascular diseases [31]. At the same time, we screened metabolites with a specific accumulation in the seed based on their absolute content, with a content greater than 1 microgram per milliliter and a difference of more than 2 times compared to the peel (Supplemental Figure S8), such as cynaroside, kaempferol, nicotiflorin, luteolin O-malonylhexoside, and other flavonoids and their derivatives, which have important roles in antioxidant and anti-inflammatory activities. Furthermore, metabolites that specifically accumulate in the peel include quinic acid and its derivatives, polyphenols, and terpenes (Figure 5C). For instance, parthenolide is a sesquiterpene lactone [32] that possesses functions such as anti-cancer [33], anti-inflammatory, and antioxidant activities [34]; clitocybin A has the functions of scavenging reactive oxygen species and anti-wrinkle [35]; picrotoxinin has neurotoxic effects and can be used as an insecticide, which may be related to the peel’s antioxidant and pest-resistant properties. Interestingly, some polyphenols and terpenes exhibit differential accumulation among different varieties of the peel (Supplemental Figure S7A–C), such as picrotoxin, cnidioside A, lansamide 4, aspergentisyl A, and quizalofop-methyl.

#### 3.4.3. Accumulation Patterns of Metabolites in the Seeds of Wampee

**Figure 6:**
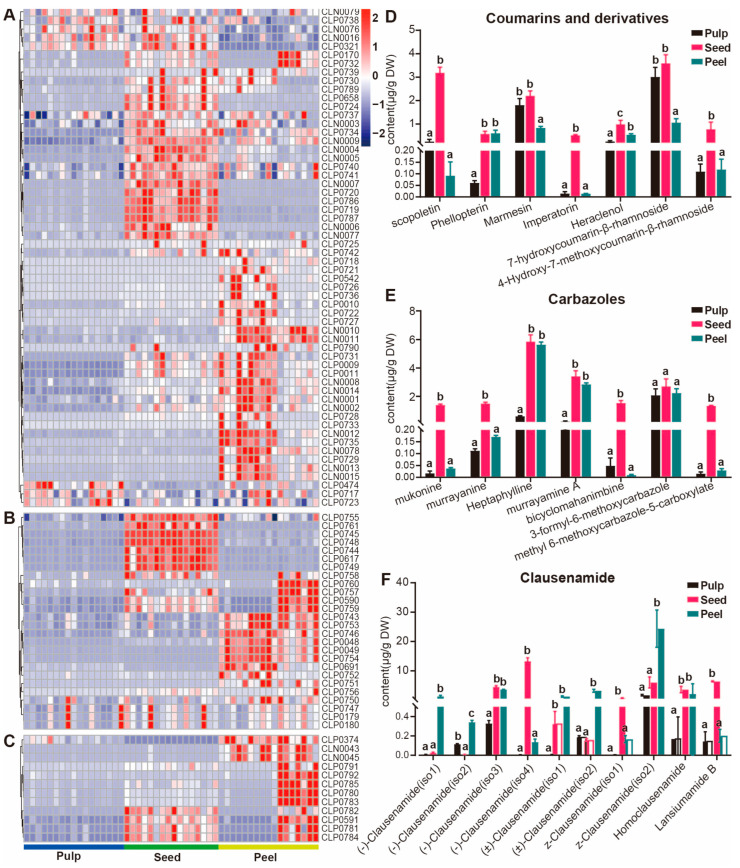
Distribution of coumarins, carbazoles, and clausenamide in different wampee fruit tissues. (**A**–**C**) Hierarchically clustered heatmap of the coumarins, carbazoles, and clausenamide metabolites from 50 wampee samples. (**D**–**F**) Bar plots showing the coumarins, carbazoles, and clausenamide content of various wampee fruit tissues, respectively. When using the abc notation to indicate significant differences, the same letter indicates no significant difference between groups (*p* > 0.05), and different letters indicate significant differences (*p* < 0.05).

Based on the hierarchical clustering analysis results of the 1286 metabolites, we conducted cluster analysis on the detected coumarins, carbazole alkaloids, and the widely reported clausenamide, which is known for its therapeutic effects on Alzheimer’s disease (Figure 6A–C). We found that these categories have certain specific accumulations in the seed and that there are significant differences in the accumulation of these metabolites among different varieties of the peel, which will provide an important reference for finding wampee fruit varieties with high medicinal value. To explore the accumulation of these metabolites in the seed, we conducted statistical analysis by calculating their absolute content (Figure 6D–F). We found that the most specifically accumulated coumarin in the seed is scopoletin, which has been found in a variety of medicinal and edible plants and plays an important role in the treatment of various diseases [36]. Coumarins such as phellopletrin, marmesin, imperatorin, and heraclenol, which are accumulated in larger amounts, have significant effects in anti-inflammatory and anti-tumor aspects [37,38,39]. Carbazole alkaloids with medicinal values such as anti-tumor, anti-inflammatory, and antioxidant effects accumulate most abundantly in the seed. For instance, heptaphylline, which can reach up to 6 µg/g in the seed, has been specifically reported to have effects in anti-malarial treatments [40]. Other carbazole alkaloids like mukonine, murrayanine, murrayamine A, and bicyclomahanimbine, although not explicitly reported, show potential for medicinal value. Among the clausenamides, (−)clausenamide has been demonstrated to improve cognitive deficits [41] and holds promise as a candidate drug for the treatment of Alzheimer’s disease and other neurodegenerative diseases [42]. The study of the accumulation pattern of important metabolites in the seed is of significant importance for the comprehensive characterization of medicinally valuable metabolites in wampee fruit.

### 3.5. Spatial Distribution Visualization of Metabolites in Wampee Fruit Based on MALDI-MSI

To explore the actual spatial distribution of differential metabolites in the three tissues of wampee fruit, we conducted precise mass measurement and mass fragment analysis of the compounds through MALDI-MSI to identify some differential metabolites in the three tissues of the wampee fruit, such as organic acids, alkaloids, coumarins, and lipids. The information on identified and some unidentified metabolites can be found in Supplemental Table S3.

**Figure 7:**
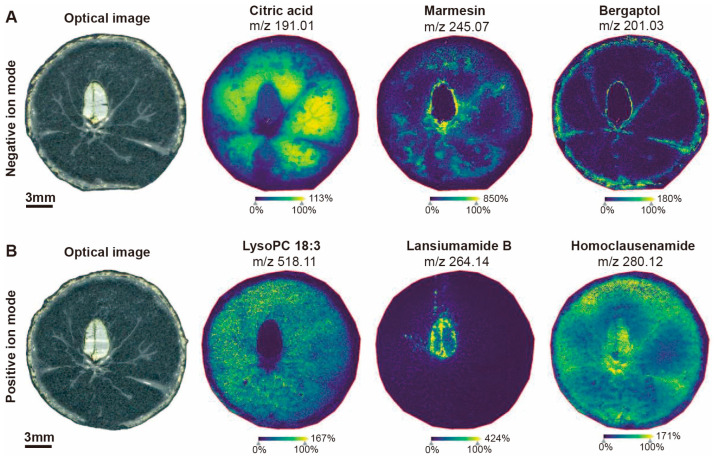
The optical image of wampee fruit slices and the spatial distribution heatmaps of various metabolites in MALDI-MSI. (**A**) The spatial distribution heatmaps of citric acid, marmesin, and bergaptol in negative ion mode. (**B**) The spatial distribution heatmaps of LysoPC 18:3, lansiumamide B, and homoclausenamide in positive ion mode. The distributions are displayed as heat maps, with the color code ranging from blue (low) to yellow (high). Images were exported from the SCiLS Lab software.

In the pulp, we identified citric acid and LysoPC 18:3, which, from a spatial distribution perspective, were found to accumulate mainly in the pulp (Figure 7), consistent with our LC–MS results (Figure 4B,D), and closely related to the nutritional value and taste of the pulp. In the peel, we identified bergaptol, which, from a spatial distribution perspective, accumulates primarily in the peel, seed coat, and placental tissue, with a higher accumulation in the peel (Figure 7), which is basically consistent with our LC–MS results (Figure 5C), but its distribution pattern is more refined and intuitive in the MALDI-MSI results. Bergaptol, as a coumarin, plays an important role in anti-inflammatory, antioxidant, and anti-tumor activities [43]. In the seed, we identified coumarin and two alkaloids; marmesin mainly accumulates in the seed coat and placental tissue (Figure 7), which further enriches our LC–MS results (Figure 6D), and marmesin has an important role in regulating angiogenesis and anti-tumor activities [44,45]. Lansiumamide B and homoclausenamide are alkaloids isolated from wampee; lansiumamide B mainly accumulates in the seed, while homoclausenamide accumulates in the seed, peel, and pulp but is more abundant in the seed (Figure 7), consistent with our LC–MS results (Figure 6F), and lansiumamide B has antifungal and anti-obesity effects [46,47]. There are also some unidentified substances whose fragment information and spatial distribution are shown in Supplemental Table S3 and Supplemental Figure S9, awaiting further identification.

### 3.6. Identify Different Varieties of Biomarker Probes

**Figure 8:**
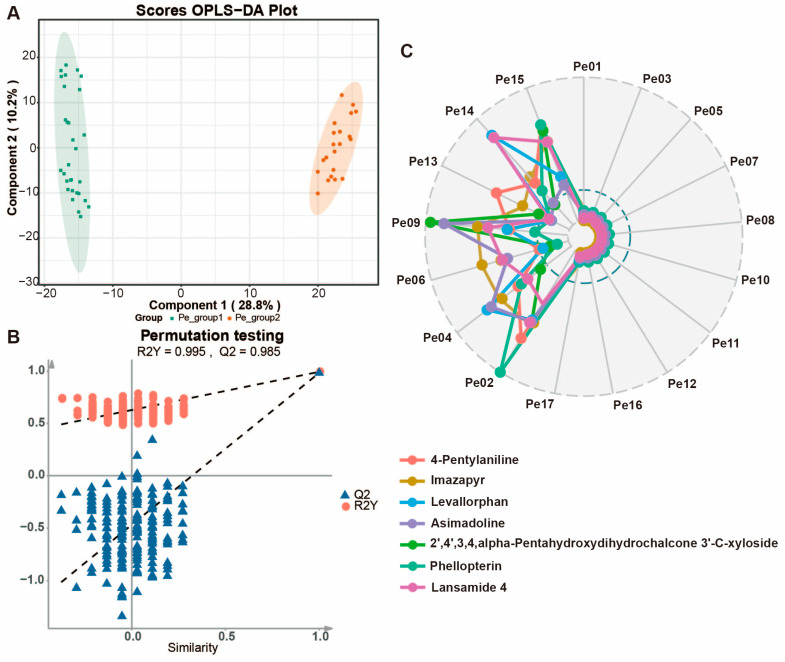
Identify different varieties of biomarker probes. (**A**) Orthogonal projections to latent structures discriminant analysis (OPLS-DA) of different wampee fruit tissues. (**B**) Permutations plot of the OPLS-DA model for the binary grouping. (**C**) Radar chart of six selected biomarkers from different categories.

Through cluster analysis, it was found that there are significant differences in the accumulation of some metabolites in different varieties of the peel, including lipids, polyphenols, terpenes, coumarins, and carbazole alkaloids (Supplemental Figure S10). To further explore the biomarkers of variety differences in wampee fruit, we conducted orthogonal projections to latent structure discriminant analysis (OPLS-DA) [48]. The OPLS-DA score plot of the scatter diagram shows significant differences between the two groups (Figure 8A). The permutation plot of OPLS-DA shows that the R2Y and Q2 values are similar and close to 1, indicating that the model has strong explanatory and predictive power for the data (Figure 8B). Therefore, the OPLS-DA model used in this study is robust and repeatable.

The results of multivariate statistical analysis indicate that the peel contributes significantly to distinguishing different varieties of wampee fruit. Through variance analysis, we screened potential biomarkers with significant changes, with *p* values ≤ 0.05, VIP values ≥ 1, and FC values > 50 or FC values < 0.02. A total of 59 potential biomarkers for different varieties of wampee fruit were identified (Supplemental Table S4). The selected differential metabolites include 27 benzene and its derivatives, 1 cinnamic acid and its derivative, 4 heterocyclic compounds, 1 indole and its derivative, 2 alkaloids, 3 coumarins, 4 lipids, 1 nucleotide and its derivative, 6 organic acids, 3 polyamines, 3 polyphenols, and 4 other metabolites. At the same time, by applying further stringent selection criteria (fold change (FC) value > 200 or FC value < 0.005), we identified 7 biomarkers (4-pentylaniline, imazapyr, levallorphan, asimadoline, 2’,4’,3,4,alpha-pentahydroxydihydrochalcone 3’-C-xyloside, phellopterin, lansamide 4) and created a radar chart (Figure 8C), which clearly divided the wampee varieties into two groups. Pe01, Pe03, Pe05, Pe07, Pe08, Pe10, Pe11, Pe12, Pe16, and Pe17 formed one group, while Pe02, Pe04, Pe06, Pe09, Pe13, Pe14, and Pe15 formed another group. The metabolites in the peels of these two groups of varieties exhibit different accumulation patterns in categories of compounds with potential for medicinal value development, such as polyphenols, terpenes, coumarins, carbazole alkaloids, and benzene and its derivatives. This is of significant reference value for exploring the medicinal value of wampee metabolites and for variety breeding.

## 4. Discussion

Wampee is native to the southern regions of China and has a long history of consumption and medicinal use in the country. The fruit of the wampee is rich in sugars, organic acids, lipids, polyphenols, flavonoids, and other compounds, providing a wealth of nutrients for the human body and possessing health benefits and medicinal value [9,49,50]. The nutritional and medicinal components and their content differences in the three tissues of the wampee fruit—peel, pulp, and seed—directly affect the commercial value and development potential of wampee, especially in areas rich in wampee germplasm resources like Yongxing. However, the current detection and comprehensive characterization of metabolites in the three tissues of the wampee fruit are insufficient, which limits the development and utilization of the wampee fruit. To gain a comprehensive understanding of the metabolic differences among the three tissues in wampee fruit, we employed non-targeted LC–MS to detect and perform PCA analysis on the peel, pulp, and seed of the wampee. The results revealed significant differences in the metabolic profiles between the three tissues (Figure 1A). To better quantify the key metabolites in wampee, we utilized the previously established widely targeted metabolomics method to construct a wampee metabolite database that includes 1286 metabolites, which is the most comprehensive wampee metabolomics database known to date, and conducted quantitative analysis on the metabolites from different varieties and different tissues collected from Yongxing Town.

Research on the metabolites of wampee has always been a major direction in wampee studies, especially in exploring the physiological functions of its metabolites. It has been shown that the ethyl acetate extract of the pericarp of wampee peel has very strong antioxidant activity and anti-cancer activity, which is higher than that of the synthetic antioxidant butylated hydroxy toluene (BHT) and the conventional anti-cancer drug cisplatin [12]. In addition, some researchers have studied the chemical composition of essential oils (EOs) from the leaves and peel of wampee and found that EOs have a wide range of antifungal activities and have the potential to be an anticandida drug [7]. A researcher studied the branch and leaves of wampee and isolated nine carbazole alkaloids, five of which showed varying degrees of resistance to tumor cells [11]. Some researchers fed polyphenol extracts of wampee leaves to rats and found that they significantly improved lipid disorders, protected the liver, and also had the effect of lowering fasting blood glucose [9]. A number of recent studies have shown that there are a large number of neuroprotective metabolites in the fruits and leaves of wampee [51], particularly dominated by clausenamide, which is expected to be a promising candidate for the treatment of Alzheimer’s disease and other neurodegenerative disorders [42], while a more detailed study has found that (−)clausenamide, but not (+)clausenamide, has an ameliorating effect on cognitive deficits [41]. However, current research on wampee is primarily focused on the overall functionality of extracts, such as wampee polyphenol extracts [9], volatile wampee essential oils [7], and wampee methanol extracts [14]. As well as the isolation and purification of metabolites from the extracts to identify new functional compounds, such as the lipid-lowering and liver-protecting metabolite zetaclausenamid [10] and neuroprotective metabolites containing a variety of new carbazole alkaloids [52,53]. In addition, there have been preliminary attempts to study the metabolites of different tissues of wampee. Ruiyi Fan and colleagues used liquid chromatography–mass spectrometry (LC–MS) for non-targeted metabolomics analysis of the leaves, barks, flowers, peels, pulps, and seeds of the wampee and identified 62 potential biomarkers to distinguish between different tissues of the wampee [54]. However, due to the small number of metabolites detected and the inability to accurately quantify them, there was no effective way to illustrate the differences between the wampee tissues, and a comprehensive characterization of this important medicinal and edible fruit could not be achieved.

In comparison, the wampee metabolite database we have established covers various categories of compounds and uses targeted metabolomics methods for precise quantification, effectively providing a comprehensive characterization of the three tissues of the wampee fruit. Additionally, our analysis found that the peel contributes the most to variety differences. By constructing an OPLS-DA model with the differences in the peel, we identified seven potential biomarkers that distinguish different varieties of wampee, providing ideas for the breeding of wampee varieties and the exploration of more substances with medicinal value. At the same time, this study also provides an important reference for the development, utilization, and protection of germplasm resources of Yongxing wampee.

In recent years, with the development of mass spectrometry imaging, an increasing number of metabolites have been characterized spatially in different species, such as rice [55], strawberries [56], and coffee beans [57]. In the case of wampee, researchers have conducted spatial mass spectrometry imaging studies on the fruit, stems, and leaves of wampee, comparing the spatial distribution of some alkaloids, coumarins, sugars, and organic acids in different tissues [58]. In comparison, our study combines MALDI-MSI with LC–MS for a more in-depth spatial imaging analysis of three tissues (peel, pulp, and seed) of the wampee fruit, which can more effectively characterize the differences in metabolites among the three tissues.

Wampee, as a fruit with both medicinal and edible properties, has great potential for development in terms of nutrition and medicinal uses. Wampee possesses effects such as antioxidant, anti-tumor, lipid lowering, liver protection, and blood sugar lowering. However, it is not very clear whether these functions are exerted in the edible parts or the medicinal parts of the fruit. This study provides a comprehensive characterization of the wampee fruit and finds that the flesh is rich in lipids, especially LPC and LPE, which can promote the digestion and absorption of lipids by the human body. Therefore, the flesh may play a role in lipid lowering and liver protection. Additionally, the flesh is rich in organic acids and amino acids, indicating that it has very high nutritional value. Moreover, the study finds that the skin is rich in flavonoids and terpenes, which typically have roles in antioxidant, anti-inflammatory, and lipid metabolism regulation. Furthermore, the core is found to be rich in carbazole alkaloids and coumarins, which play important roles in anti-tumor and anti-cancer effects and are also important categories for exploring medicinal value metabolites. Thus, this study characterizes the functions of different parts of the wampee fruit, providing a basis for the refined processing of wampee fruit. At the same time, this study characterizes the nutritional and medicinal values of wampee fruit, offering a reference for the exploration of metabolites with health care functions and medicinal value. It is of great significance for the development and utilization of wampee fruit.

In summary, we have comprehensively characterized the metabolites in the three tissues of multiple varieties of wampee fruit using the broad-targeted metabolomics approach with LC–MS and combined this with MALDI-MSI for an intuitive display of the spatial distribution of wampee fruit metabolites. These efforts provide significant reference value for the development and utilization of wampee.

## 5. Conclusions

In this study, by combining LC–MS and MALDI-MSI, we conducted a systematic detection and comprehensive characterization of metabolites in the three tissues of fruits from 17 wampee varieties in Yongxing Town. We found that the pulp is rich in lipids, amino acids, and organic acids, which may be related to its nutritional value; the peel is rich in flavonoids, quinic acid, polyphenols, and terpenes, which may be related to its antioxidant and anti-inflammatory properties; and the seed is rich in carbazole alkaloids, coumarins, and clausenamides, which may be related to its medicinal value. Additionally, we found that the peel contributes the most to the differences between varieties, especially some compounds with medicinal potential. All of this provides reference value for the development and utilization of Yongxing wampee, as well as for variety breeding and the protection of germplasm resources.

## Data Availability

The original contributions presented in the study are included in the article/Supplementary Materials, further inquiries can be directed to the corresponding author.

## Supplementary Materials

The following supporting information can be downloaded at: https://www.mdpi.com/article/10.3390/foods13193092/s1, Figure S1. Photos of Wampee Fruit Varieties Involved in This Study. Figure S2. Detection and identification of specific catechin, gallocatechin, and scopoletin metabolite signals by ESI-Q-Exactive LC-MS/MS. Figure S3. Assessment of sample repeatability and Venn diagram of the three tissues. Figure S4. Volcano plots of metabolites between pairwise comparisons of the three types of tissues. Figure S5. Distribution of glycerophospholipids in different wampee tissues. Figure S6. Distribution of flavonoid subclasses in different wampee tissues. Figure S7. Distribution of polyphenols, terpenes, quinate and its derivatives in different wampee tissues. Figure S8. Bar plots showing the flavonoids that are more abundant in the seeds of wampee. Figure S9. The optical image of wampee fruit slices and the spatial distribution heatmaps of various metabolites in MALDI-MSI. Figure S10. Hierarchically clustered heatmap of the 345 annotated metabolites from 17 wampee peel samples. Table S1. The information of wampee variety used in this study. Table S2. Selected partial differential metabolites exist between and among the three tissues of the wampee fruit. Table S3. Identification of metabolites in wampee by MALDI-MS analysis. Table S4. Identification of potential biomarkers for different varieties of wampee.
