# Supplementary material for: Insights into Tissue-Specific Specialized Metabolism in Wampee (Clausena lansium (Lour.) Skeels) Varieties
Source: Foods. 2024 Sep 27;13(19):3092. doi: 10.3390/foods13193092 (PMC11475070; doi:10.3390/foods13193092)
Supplement: Supplementary file 1 [file foods-13-03092-s001.zip › Supplementary figures and tables.pdf]

Supplementary Figures and Tables

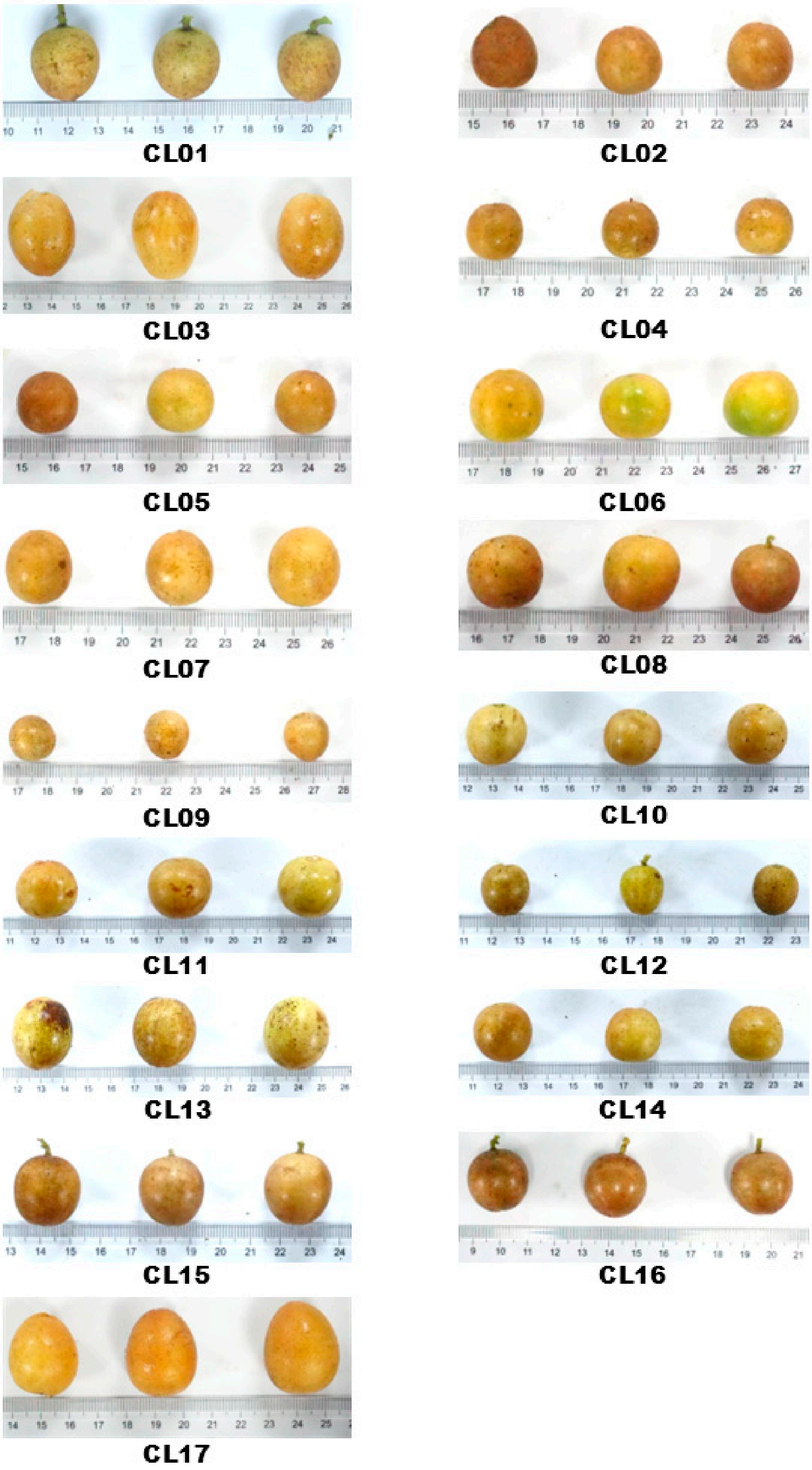

Supplementary Figure S1. Photos of Wampee Fruit Varieties Involved in This Study

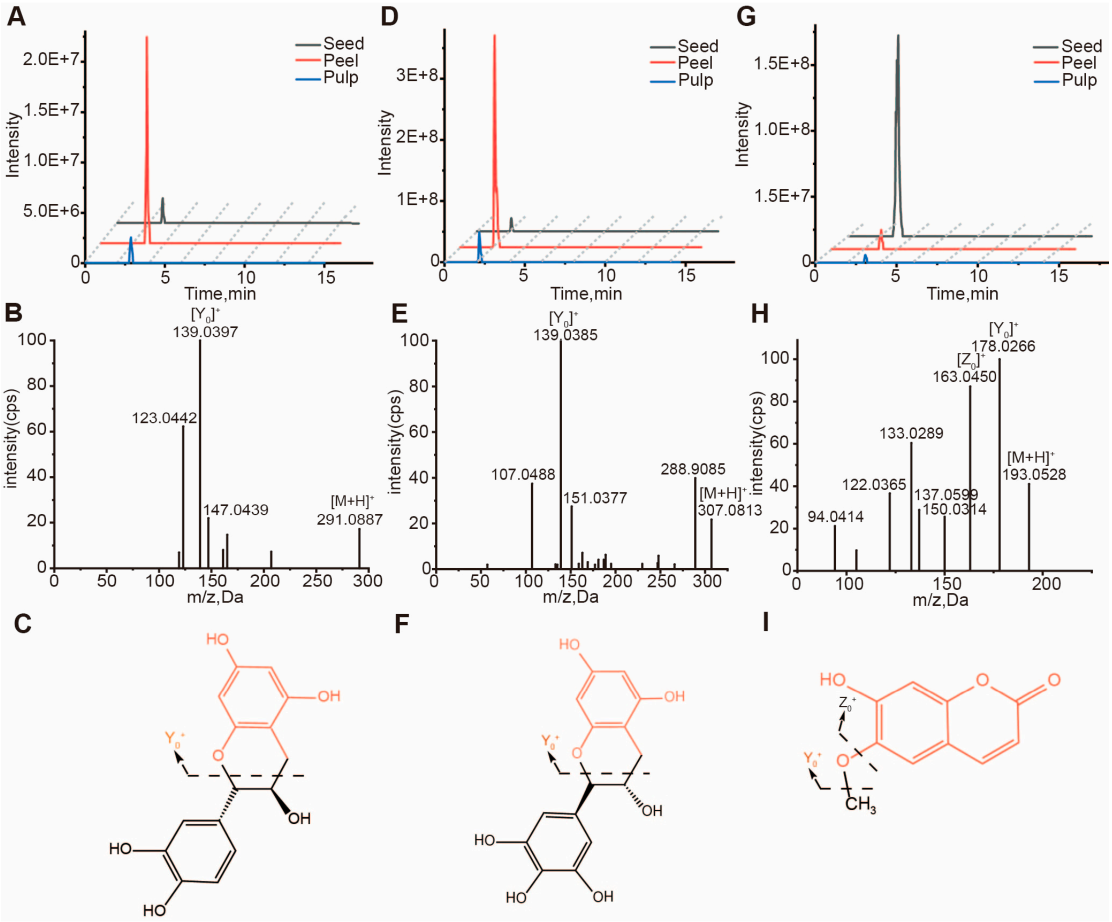

Supplementary Figure S2. Detection and identification of specific catechin, gallocatechin, and scopoletin metabolite signals by ESI-Q-Exactive LC-MS/MS.

(A) EIC (extracted ion chromatogram) of CLP0766 at 2.93 min.

(B) MS/MS spectra of CLP0766 at m/z 291.0887, and the metabolite was identified as catechin.

(C) The molecular structure of the catechin and its general fragmentation rules.

(D) EIC (extracted ion chromatogram) of CLP1109 at 2.15 min.

(E) MS/MS spectra of CLP1109 at m/z 307.0813, and the metabolite was identified as gallocatechin.

(F) The molecular structure of gallocatechin and its general fragmentation rules.

(G) EIC (extracted ion chromatogram) of CLP0720 at 3.812 min.

(H) MS/MS spectra of CLP0720 at m/z 193.0528, and the metabolite was identified as scopoletin.

(I) The molecular structure of scopoletin and its general fragmentation rules.

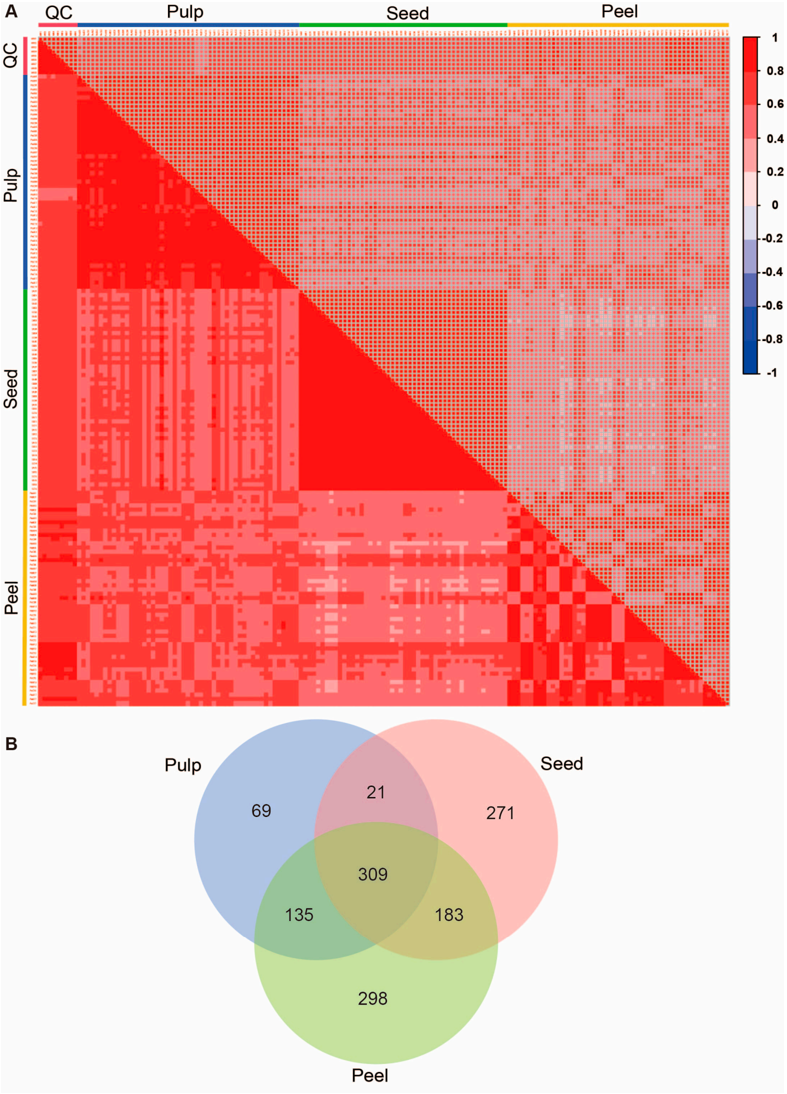

Supplementary Figure S3. Assessment of sample repeatability and Venn diagram of the three tissues.

(A) Sample repeatability assessment for 17 varieties with triplicate tissues, three replicates, and quality control samples.

(B) Venn diagram illustrating the relationships among three different tissues.

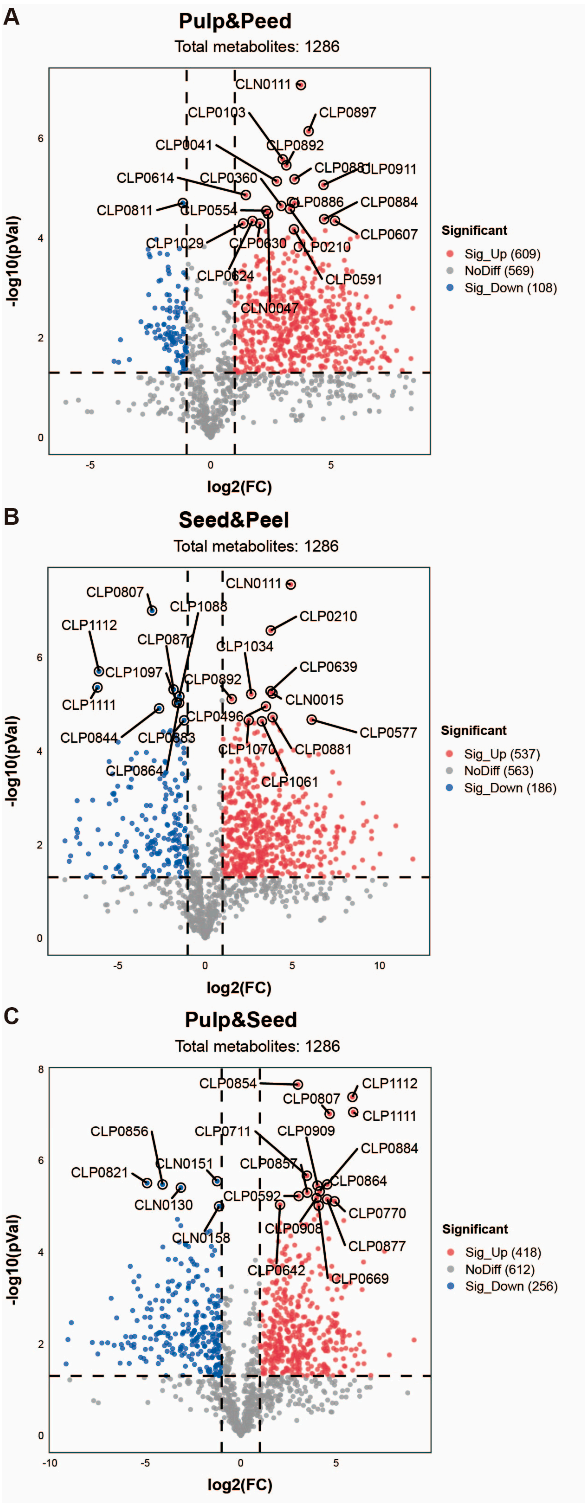

Supplementary Figure S4. Volcano plots of metabolites between pairwise comparisons of the three types of tissues.

(A) Volcano plots of differential metabolites between the pulp and peel of Wampee Fruit.

(B) Volcano plots of differential metabolites between the seed and peel of Wampee Fruit.

(C) Volcano plots of differential metabolites between the pulp and seed of Wampee Fruit.

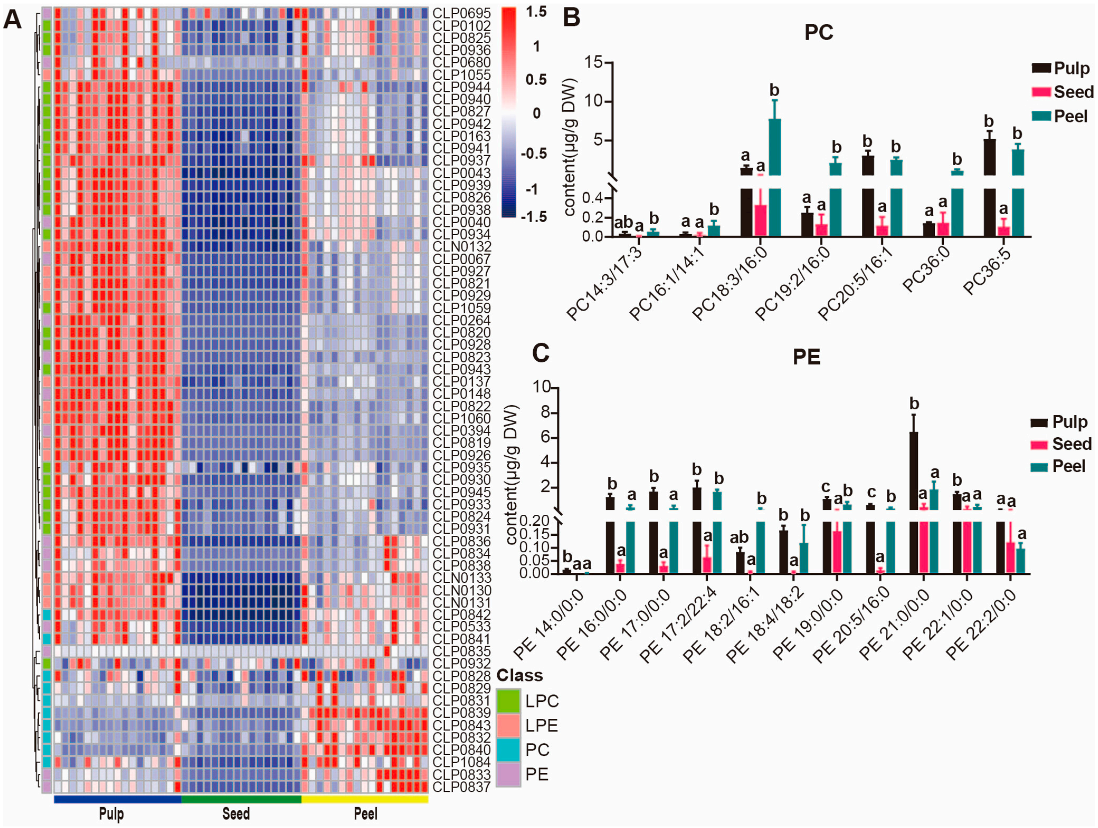

Supplementary Figure S5. Distribution of glycerophospholipids in different wampee tissues.

(A) Heatmap visualization of metabolome data of glycerophospholipids in 50 samples. Expression data were Z-score standardized.

(B-C) Bar plots showing the contents of PC (B) and PE (C) content of various wampee tissues.

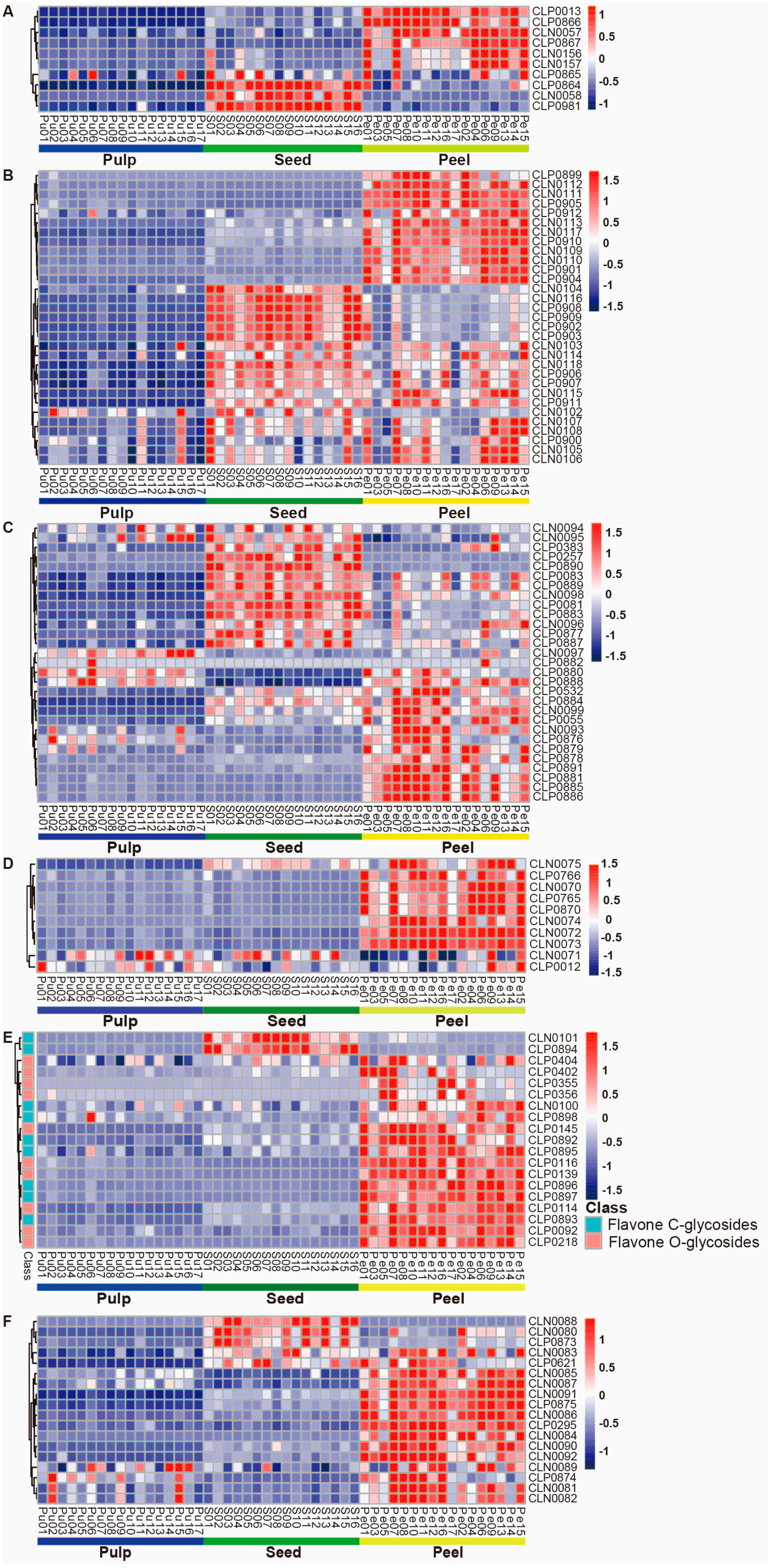

A
CLP0013
CLP0866
CLN0057
CLP0867
CLN0156
CLN0157
CLP0865
CLP0864
CLN0058
CLP0981
1
0.5
0
-0.5
-1
Pulp
Seed
Peel
B
CLP0899
CLN0112
CLN0111
CLP0905
CLP0912
CLN0113
CLN0117
CLP0910
CLN0109
CLN0110
CLP0901
CLP0904
CLN0104
CLN0116
CLP0908
CLP0909
CLP0902
CLP0903
CLN0103
CLN0114
CLN0118
CLP0906
CLP0907
CLN0115
CLP0911
CLN0102
CLN0107
CLN0108
CLP0900
CLN0105
CLN0106
1.5
1
0.5
0
-0.5
-1
-1.5
Pulp
Seed
Peel
C
CLN0094
CLN0095
CLP0383
CLP0257
CLP0890
CLP0083
CLP0889
CLN0098
CLP0081
CLP0883
CLN0096
CLP0877
CLP0887
CLN0097
CLP0882
CLP0880
CLP0888
CLP0532
CLP0884
CLN0099
CLP0055
CLN0093
CLP0876
CLP0879
CLP0878
CLP0891
CLP0881
CLP0885
CLP0886
1.5
1
0.5
0
-0.5
-1
-1.5
Pulp
Seed
Peel
D
CLN0075
CLP0766
CLN0070
CLP0765
CLP0870
CLN0074
CLN0072
CLN0073
CLN0071
CLP0012
1.5
1
0.5
0
-0.5
-1
-1.5
Pulp
Seed
Peel
E
CLN0101
CLP0894
CLP0404
CLP0402
CLP0355
CLP0356
CLN0100
CLP0898
CLP0145
CLP0892
CLP0895
CLP0116
CLP0139
CLP0896
CLP0897
CLP0114
CLP0893
CLP0092
CLP0218
1.5
1
0.5
0
-0.5
-1
-1.5
Class
Flavone C-glycosides
Flavone O-glycosides
Class
Pulp
Seed
Peel
F
CLN0088
CLN0080
CLP0873
CLN0083
CLP0621
CLN0085
CLN0087
CLN0091
CLP0875
CLN0086
CLP0295
CLN0084
CLN0090
CLN0092
CLN0089
CLP0874
CLN0081
CLN0082
1
0.5
0
-0.5
-1
Pulp
Seed
Peel

Supplementary Figure S6. Distribution of flavonoid subclasses in different wampee tissues.

Heatmap visualization of metabolome data of anthocyanins **(A)**, flavonol **(B)**, flavone **(C)**, catechin and derivatives **(D)**, flavone glycosides **(E)**, and flavanone **(F)** in 50 samples. Expression data were Z-score standardized.

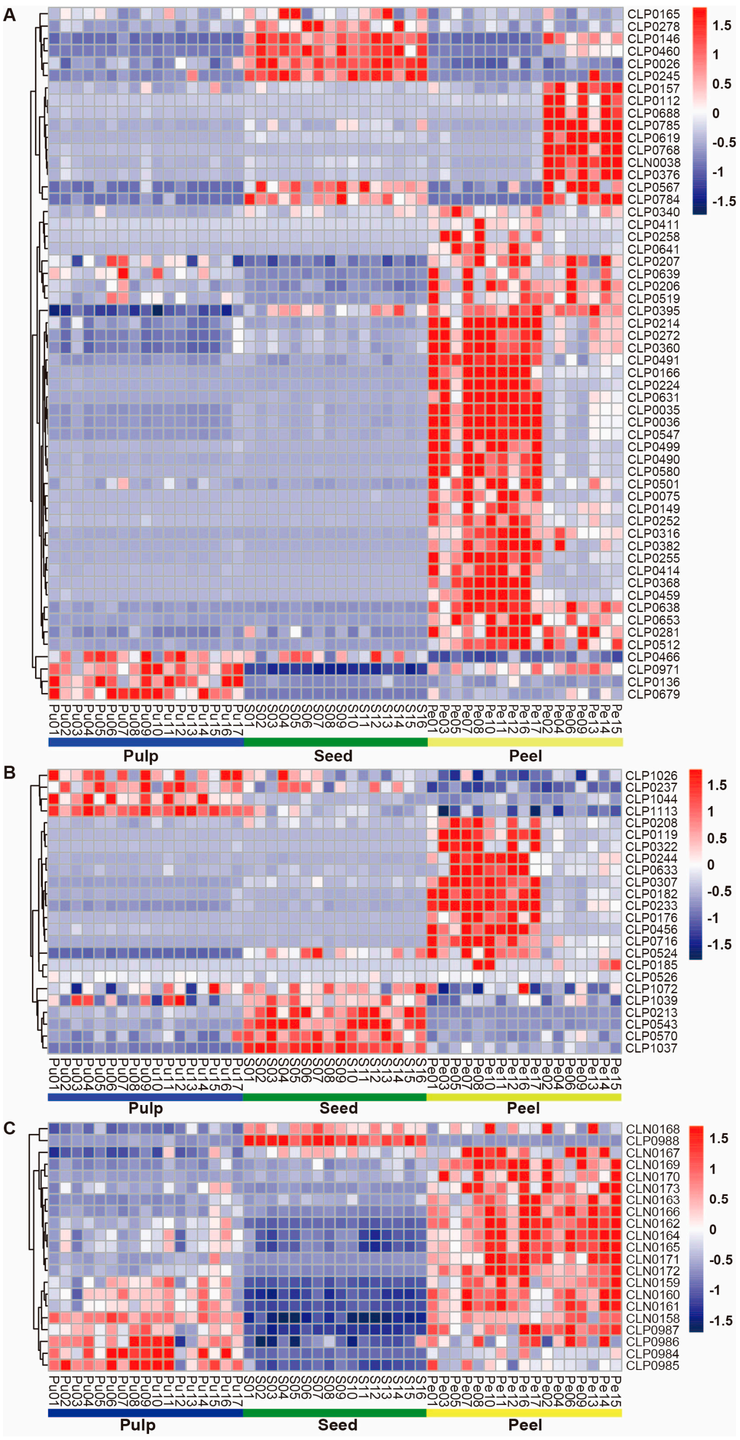

Supplementary Figure S7. Distribution of polyphenols, terpenes, quinate and its derivatives in different wampee tissues.

Heatmap visualization of metabolome data of polyphenol **(A)**, terpenes **(B)**, quinate and its derivatives **(C)** in 50 samples. Expression data were Z-score standardized.

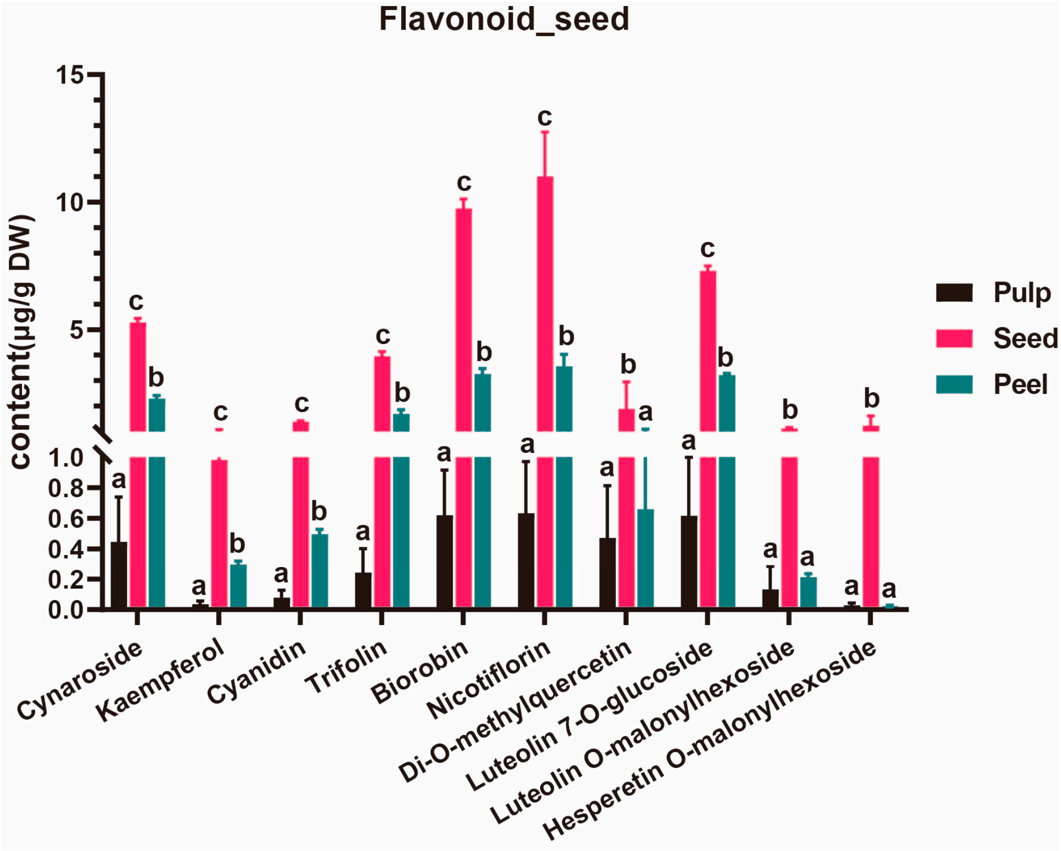

Supplementary Figure S8. Bar plots showing the flavonoids that are more abundant in the seeds of wampee. When using the abc notation to indicate significant differences, the same letter indicates no significant difference between groups ($p > 0.05$), and different letters indicate significant differences ($p < 0.05$).

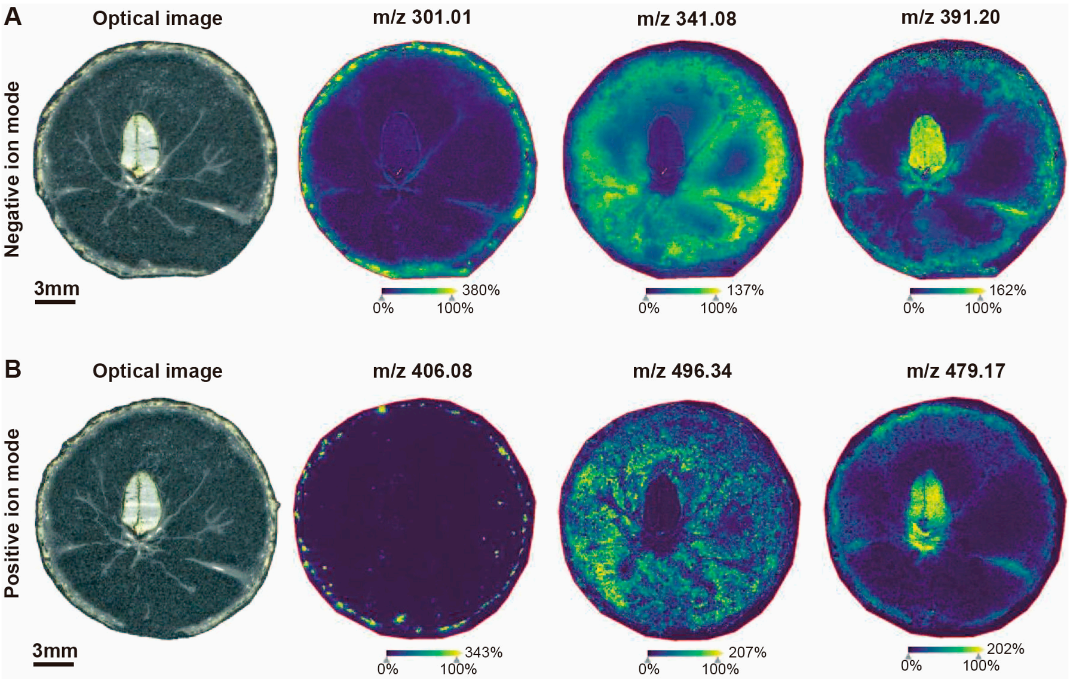

Supplementary Figure S9. The optical image of wampee fruit slices and the spatial distribution heatmaps of various metabolites in MALDI-MSI.

(A) The spatial distribution heatmaps of the unidentified substances with m/z values of 301.01, 341.08, and 391.20 in negative ion mode.

(B) The spatial distribution heatmaps of the unidentified substances with m/z values of 406.08, 496.34, and 479.17 in positive ion mode.

The distributions are displayed as heat maps, with the color code ranging from blue (low) to yellow (high). Images were exported from the SCiLS Lab software.

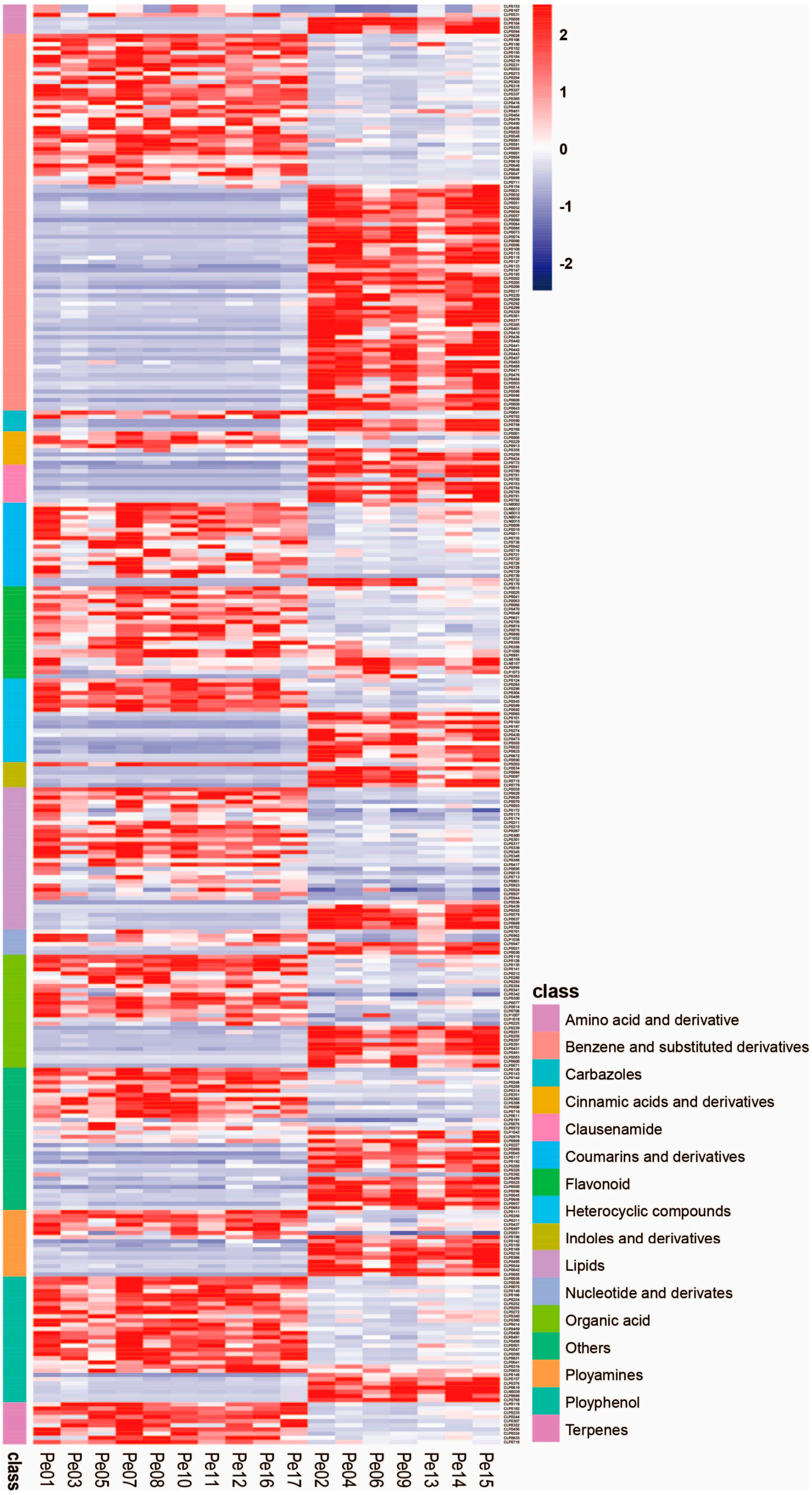

Supplementary Figure S10. Hierarchically clustered heatmap of the 345 annotated metabolites from 17 wampee peel samples.

Supplementary Table S1. The information of wampee variety used in this study.

Supplementary Table S2. Selected partial differential metabolites exist between and among the three tissues of the wampee fruit.

Supplementary Table S3. Identification of metabolites in wampee by MALDI-MS analysis.

Supplementary Table S4. Identification of potential biomarkers for different varieties of wampee.
